# A priority based energy harvesting scheme for charging embedded sensor nodes in wireless body area networks

**DOI:** 10.1371/journal.pone.0214716

**Published:** 2019-04-22

**Authors:** Md Khurram Monir Rabby, Mohammad Shah Alam, MST Shamim Ara Shawkat

**Affiliations:** 1 Institute of Information and Communication Technology (IICT), Bangladesh University of Engineering and Technology (BUET), Dhaka, Bangladesh; 2 Department of Computer Science, Tennessee Technological University, Cookeville, United States of America; 3 Department of EECS, University of Tennessee, Knoxville, United States of America; Northeast Electric Power University, CHINA

## Abstract

This research work proposes a novel priority aware schedule based charging algorithm that uses wireless power transfer (WPT) technique in order to charge embedded sensor nodes (SNs) in a wireless body area network (WBAN). Implanted sensor nodes in WBANs require energy for both information extraction and data transmission to the remote controller unit. Thus, energy shortage of these SNs deteriorates due to the data transmission process of the patient health monitoring system. However, continuous operation by means of electromagnetic induction for energy harvesting, obtained from ambient sources, reduces the overall efficiency of the primary unit. With this paradigm in sight, an algorithm demonstrating the modeling of a priority-based mechanism is proposed in order to ensure proper sensor voltage level and to reduce the transmission losses. Medium access control (MAC) protocols are used for inductive powering from the primary unit to the secondary unit in a collision-free centralized scheduling scheme. Therefore, the proposed wireless charging algorithm for implanted SNs in WBAN is designed as per carrier sense multiple access with collision avoidance (CSMA/CA) technique. Because of this, the overall power consumption of SNs for certain operation periods, successful charging probabilities for multiple SNs, and instantaneous power requirements are considered as key performance measures of analysis. It is assumed that proper energy storage in both transmitters and receivers can handle channel interference and traffic contention. Simulation results verify that a significant reduction in power consumption for the proposed priority aware algorithm will maintain almost similar output. For this reason, saturating class—C as well as class—E driver circuits have been used to justify the performance in two different circuit topologies. Effects of priority with respect to the full charge period have also been observed for the multi-node system. Furthermore, from performance analysis, it has been demonstrated that the scheduling scheme causes both single MOSFET composed saturating class—C and L_*choke*_ modeled class—E associated driver circuits to be considerably more loss efficient than corresponding existing ones.

## 1 Introduction

In recent years, researchers have been trying to use different types of sensors in a wireless body area network (WBAN) to achieve an earlier continuous diagnosis, instead of letting health problems go undetected [1–3]. At present, people with sustained paralysis or limb amputations learn to control a robotic arm by implanted brain sensors through brain-computer interfaces (BCI) [4]. Moreover, wireless sensors are implanted to mount immediately on the skin in order to pick up vital signs of temperature, pulse, and breathing rate. Besides that, researchers are also applying the new technology for implanting sensors into more sensitive areas of the human body, such as brain and spinal cord, in order to release epilepsy drugs [5]. However, charging the existing battery-powered implanted sensor nodes is very challenging and the replacement from the inside of the human body is very inconvenient, if not completely impossible [6]. In such cases, wireless power transfer (WPT) is a viable solution, although energy consumption, overall efficiency and voltage regulation of a fixed secondary circuit will be challenging tasks. Charging all nodes continuously, including implanted sensors, from a controller (primary circuit) will restrict the patient’s mobility, as well as cause energy wastage. As a result, in this research, two different cases saturating class—C as well as class—E driver circuits are considered as a part of primary for charging implanted sensor nodes as secondary. Though two circuit driver classes, namely saturating class—C as well as class—E, are implemented for generating inductive links to charge implanted sensor nodes, their overall efficiency levels are not found to be satisfactory yet [6]. Therefore, comparative analysis for on-demand wireless energy extraction of a priority aware scheduling algorithm with satisfactory energy utilization in order to improve overall performance for embedded sensor nodes is not going to be discussed.

## 2 Related works

Wireless body area network (WBAN) has emerged as a solution of the traditional health care system [3]. It provides both flexibility and cost-saving options for new applications, under the umbrella of wireless communication domain. For this reason, its application is found both in medical and non-medical functioning such as sports, entertainment, health and emergency situations [7–10]. It has a significant impact on multi-wireless networks due to the range in applications from environmental to border security, and from structural monitoring to human health supervision [11–18]. These multi-hop networks consist of sensor nodes (SNs) and ensure vital wireless connection by extracting, processing, and transmitting the real-time data in critical operations [19–35]. It permits continuous supervision of physiological parameters by allowing for greater mobility and flexibility of a patient. Wireless sensor nodes (WSNs) of WBANs change the requirement of patients that tend to spend long periods of time in the hospital for monitoring [36, 37]. It simplifies and improves reliability in remote uninterrupted communication with patients under natural physiological states, without constraining their normal activities at hospital and homecare environments [3]. The portability and monitoring independent facility of real-time feedback in critical health conditions, allow for the transmission of data in a non-invasive manner for earlier detection of abnormality [36, 38]. Therefore, it has been a key issue within e-health to provide unobtrusive ambulatory services from a physician in order to ensure the quality of life for patients in many situations [1, 36, 38, 39].

All these facilities of WBAN make researchers even more interested in this terminology. WBAN is a network of low-power, intelligent, micro and nano-technology sensors, actuators and gateway nodes that are small enough to be implanted or connected in or around the human body [3, 7, 37]. These actuators and sensors are placed on/inside the patient body and connected wirelessly. The purpose of those in or on-body sensor nodes (named as WSNs) is to accumulate physiological information and transmit this data to an external database server. The gateway nodes acting as telecommunication network help to store collected data on the user’s personal digital assistant (PDA) and transfer this data to a remote computer [36].

Present researchers think about different system design and implementation opportunities that improve the overall performance of WBAN. The major challenges they need to overcome are the delay minimization, throughput maximization, reduction of energy consumption for communication purpose, and maximization of network lifetime [3, 40, 41]. All these constraints are related to the transmitter, which balances time sensitivity and importance of buffered data, in order to control frame overhead, idle listening and frame collisions. According to IEEE 802.15.6, beacon communication mode (where the hub works as a supervisor for synchronization and resource allocation) and non-beacon communication mode (where there are polling and scheduled or unscheduled allocations for resource distribution) are both two acceptable procedures that handle data directly for collision avoidance and save energy indirectly, as per the system requirement [42]. However, without a demand-based power-efficient infrastructure for normal and emergency traffic, the presence of contention and channel interference in the wireless network affect the communication quality. Increasing the transmitter power improves data handling capacity, allowing for better communication [2, 39, 43, 44]. Subsequently, depending on different sensor capabilities, resources, and the level of both intelligence and sampling rates, dynamic power handling is the key challenge in WBANs applications [1, 37, 43]. To handle the dynamic power challenge, WBANs require channel resources to be shared. Thus, the exclusive access phase (EAP) has been implemented in different user priorities and access methods to improve performance (i.e. throughput normalization, energy consumption, and service time allocation between mean frames) [42]. Nonetheless, EAP causes a delay for nonpriority nodes by both reducing the overall system throughput and consuming extra energy per frame. Therefore, research in WBANs tends to be toward scheduling or priority-based scheduling techniques, for the comparative better patient monitoring system. These techniques have been discussed in order to mitigate the mutual interference between nodes for both the idle listening and contention complexity problems [45, 46]. In the same concept, the traffic-aware dynamic MAC protocol (TD-MAC) has been used for controlling the wake-up period in order to save the energy consumption from overhearing, idle listening, unnecessary beacon transmission, and collision [47]. The low-delay, traffic-adaptive MAC (LDTA-MAC) priority has been introduced to address the dynamic time slot allocation in energy consumption [48, 49]. In the traffic-adaptive MAC protocol [50], wakeup tables have been proposed in order to make the transmission schedules of nodes. A heart rhythm synchronization used for reducing energy costs has been discussed in the heartbeat driven MAC protocol (H-MAC) [51]. Besides, packets priority has been discussed for QoS to the packets according to their traffic priority level and load-adaptive MAC (PLA-MAC) protocol [52]. Priority-guaranteed MAC protocol (PMAC) has been discussed in [53] for significant data packet accommodation. A priority has also been imposed on different nodes based on the criticality both data packets and the energy resources [54]. In the priority-based adaptive MAC (PA-MAC) protocol, time slot allocation has been selected dynamically, following the traffic priority (i.e. channel status, energy consumption and transmission time) in unlicensed bands [55]. A prioritized resource allocation (PRA) algorithm has also been proposed, based upon service priority for both network performance improvement and device compatibility enhancement, among various devices as per IEEE 802.15.6 [56]. An adaptive time division multiple access (TDMA) scheme has been used for traffic scheduling in order to provide multiple access efficiency and improve the quality of service. However, task-related charging issues have not been taken into consideration in these scheduling schemes. Therefore, all these issues convert into the resource-constrained problem, which means battery issues for very small and distributed SNs. Frequent battery replacement to extend charge period has been discussed in [43]. This replacement or recharging procedure causes not only system operation to be slow and expensive but also causes a decrease in network performance [11–18]. On the contrary, battery-powered sensor nodes have small and limited processing capabilities. Non-rechargeable batteries cause the serious problem of spending significant power for information processing purposes. Accordingly, variation in energy depletion of nodes consequentially hampers the overall efficiency and lifetime of the network [57]. To avoid the complexity of accessing sensors and changing the associated batteries, researchers are putting effort towards the autonomous duty cycling and power saving schemes in different protocol layers of the stack, like medium access control (MAC), time division multiple access (TDMA) etc [58–67]. Nevertheless, the application of different protocols can handle the energy resource issue for a certain period. To overcome this problem in WSNs, energy harvesting (EH) endows the capability of exploiting from surrounding energy sources. It is possible to enable WSNs to last potentially forever for future use [11–18]. Both the device and storage of energy need processing time in order to harvest energy from the environment. Therefore, processor scheduling is needed for the continuation of work tasks. A dynamic scheduling algorithm has been proposed, based on voltage and frequency scaling pertaining to energy harvesting embedded systems. This algorithm concentrates free time, in order to harvest energy [68]. Similarly, both the earliest-deadline-first (EDF) scheduling and least-leisure-time-first (LSF) are two algorithms used for task scheduling, based on order assignment and priority respectively. Although, these algorithms are dealing with order and priority level, thus, they are not appropriate to use in real time energy sensitive devices for the energy consumption of peripherals [69]. Provided that energy saving depends highly upon on the shared devices, the task set, and the power requirement. Energy-aware EDF (EA-EDF), enhanced energy-aware EDF (EEA-EDF), and slack utilization for reduced energy (SURE) are three well-known algorithms used for their dynamic power management systems. Although the algorithms perform well in discrete time, low power, and state switching devices, they are not capable of performing well in a continuous time system operation [70]. Very recently, machine learning comes as an alternative to offline data collection, for the batch processing of eventual insight actions. Although data processing is fast and action generation is quick, actions are required to be taken offline, and effects cannot be incorporated back immediately into the learning process. To overcome this problem, instead of considering an “open-loop” system, a closed-loop system has been proposed which is termed as an adaptive machine learning (AML). In WBANs, data streaming happens online. In order to handle online data streaming, AML follows a “recursive” technique which often makes the process complex in a big solution space. Another issue in AML is that it often requires offline training in order to make the system adaptive for multiple patients. Since every person is unique, a large training data set needs to be created, which may be inappropriate for special cases. Although the importance of machine learning or AML in WBANs can be a good solution for dynamic power management, research is still going on to generate an algorithm in machine learning in order to cooperate with these issues [71–75]. For schedule node charging, radio frequency (RF) requires a higher broadcast power of information transmission, which in turn causes additional interference in data communication [66, 76]. Therefore, electromagnetic induction as wireless power transfer (WPT) could be an alternative idea to meet the challenges of the energy issues in WBAN [77]. On the other hand, the maximization of communication to overcome system interference has not been solved. For the time being, researchers solve this interference problem by power minimization. For this reason, the power control model has been used to account for both data packet streaming and channel interference measures. Meanwhile, in [78], another challenge of SNs is discussed as the adaptive connection latency, which is not solved due to the maximum avoidance of interference.

WBAN is an ongoing research process which can provide better service options to the user end, especially for the patient. Therefore, it is possible to monitor the human body internal sensitive organs functionality through SNs using WBANs technology. To keep track of the functionality of those organs properly, existing research has been done on scheduling or priority aware scheduling techniques, for the collision avoidance data transmission in WBAN. However, the internal energy of these SNs is an important issue when achieving an uninterrupted data transmission. Some of the SNs are irreversible, which will die out due to the lackings of enough energy. In that case, the patient has to go through a complicated operation procedure once again for the replacement of these SNs. Considering the energy limitation, there are some research works in the energy saving scheme that prolongs the operation lifetime of SNs in WBANs, however, none of them are everlasting. Moreover, there are many dynamic power/energy management algorithms for charging embedded SNs, but none of them have been designed with the consideration to the WPT technique in WBAN. WPT could be a viable solution to charge in/on body SNs in WBANs on the requirement basis of continuous, long-term operation. All these problems motivate the study and model of such a scheme from the circuit level, that can charge the resources without causing any problem to the patient, while at the same time minimizing interference and controlling the latency of sensor nodes in a scheduled way.

## 3 Research objectives

In this research study, the energy extraction technique is identified as a task scheduling problem for charging embedded SNs in WBANs. A task scheduling has to satisfy the constraints of order and time requirement. Therefore, a priority aware scheduling-based scheme is proposed for energizing implanted SNs in WBAN. The prioritized charge scheduling allocation algorithm employs the voltage level information to charge SNs based on WBANs service priority. The suggested approach is implemented using inductive WPT circuitry constructed by a saturating class—C and a class—E driver circuits respectively. In this study, the network is assumed non-saturation condition to avoid unfair channel access. Closure placement of sensor nodes in WBAN causes the power circuitry to be selected for limited range, designed voltage, and current rating. The priority aware scheduling scheme is implemented in the primary circuitry after designing the electronic link for an on request-based power demand from a secondary unit. The main purpose of the secondary nodes is to transfer surrounding information into the central decision unit. Thus, a cut-off voltage rating is tried to maintain within 10% of the nominal sensor operation rating in order to ensure a continuous operative mode. In this design, the controller unit is considered as the main monitoring circuit device for analyzing received information from different SNs. In addition to that, it gives necessary instruction through the controller in the primary in order to apply the priority aware algorithm through the MAC protocol, in order to keep the nominal designed rating in the secondary. In a simulation environment, multi-sensor node-based circuitry is considered as test systems in order to justify the priority aware scheduling algorithm for the dedicated WPT in WBANs. For this reason, both an instantaneous power requirement and overall power consumption of SNs within a certain operation period have been used as parameters of interest. In addition to that, successful charging probability for multiple SNs have been used to show the classification as per priority for charging. A review of the empirical search of the relevant literature yielded that priority aware based scheduling, using WPT in order to charge SNs in WBANs has not been mentioned previously. That is why simulation results show the comparison between a priority based scheme and an existing technique (non-priority based) in continuous power consumption for single, a double MOSFET oriented saturating class—C, and an L_*choke*_ modeled class—E drivers’ associated sensor nodes. It is justified that the proposed driver circuits are more efficient for the suggested priority aware scheduling scheme than the existing technique. The focus of this research work is to investigate the possibility of charging the sensor nodes on a priority basis using wireless power transfer technique. To the best of our knowledge, charging sensor nodes in WBAN environment based on the node’s priority has not been studied before, especially from the perspective of wireless power transfer (WPT).

The remaining part of this research work is as follows. WBANs system modeling for multiple sensor nodes in the human body connected through MAC control are presented in section 4. In section 5, scheduling technique for charging WSNs in WBANs is discussed. Moreover, the priority aware scheduling algorithm is designed as per a priority aware CSMA/CA technique (IEEE 802.15.4) for charging WSNs of WBANs in section 6. A proposed priority scheme based simulation results and comparative analysis have been justified in section 7. Finally, concluding remarks as well as a future perspective of this research are given in section 8.

## 4 Wireless body area network based system modeling

Implanted SNs are designed for wireless communication with the limited energy in WBAN. The purpose of this wireless communication is to render convenience of the patient and transfer information from within the human body to a coordinator unit for monitoring the transmitted data. For this reason, a coordinator unit called the controller has been connected wirelessly with these embedded sensor nodes. In this analysis, this transferred information has been classified into two parts: the environmental and the physical state of the human body, and the voltage level information about the embedded sensor nodes inside the human body. Like prototypical WBAN, data extraction from implanted SNs is an issue for the transmitter within a certain period of time. The controller unit maintains this time interval. In addition to that, required power information will also be monitored by the controller unit. Therefore, proposed scheduling will consider any voltage below the threshold level and transmit necessary commands to the corresponding circuit in order to start the energizing process according to the algorithm. In this study, different frequency levels have been counted in order to avoid collision between the data transmission and the inductive link setup from the primary to the secondary circuit. Moreover, in the controller circuit, a data transmission monitoring procedure is implemented as per the proposed priority aware CSMA/CA technique (IEEE 802.15.4).

**Fig 1:**
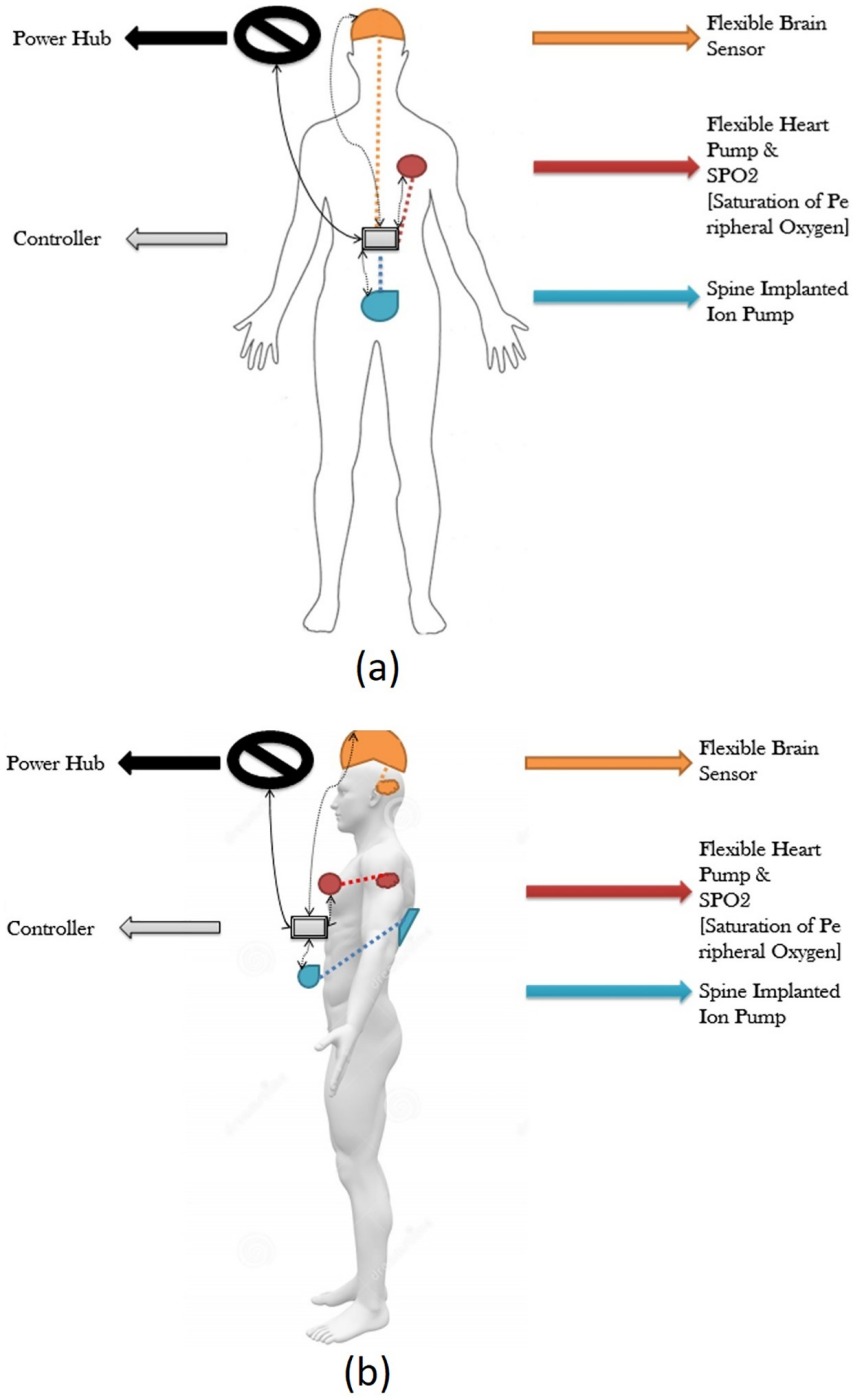
Possible sensor nodes in system modeling of WBANs [79]. (a) Front view of human body (b) Side/lateral view of human body.

Modeling of WBANs is shown in Fig 1. In this system, three embedded sensor nodes, considered under the same WBANs, are connected to each other with a controller unit. An external power source named as the power hub provides energy through a wired media to the controller unit to ensure uninterrupted power supply. Here, three embedded sensor nodes act as the heart pump with SPO2, a flexible brain sensor, and a spline embedded ion pump. In Fig 1b, a dedicated primary circuit has been shown for each sensor placed immediately on the human body. The reason for the primary unit is to ensure an essential inductive connection in order to replenish the power requirement of the embedded sensor nodes. All these primary circuits are energized by small wearable battery sources and are wirelessly connected with the controller unit following the IEEE 802.15.4 protocol. On the contrary, a secondary implanted device is directly associated with the on-body primary unit for medical data analysis purposes following the contention free CSMA/CA method. Therefore, the controller unit not only monitors collected data from inside the human body but also ensures the voltage level of the primary circuit and the power requirement of the embedded secondary circuit.

To monitor the present health condition of a patient, there are many in-body sensors required to charge periodically in order to receive data in a regular way regarding the health situation. Therefore, the charging schedule is required to be adjusted in a priority aware scheme, in order to give the preference for better performance. Embedded in-body and on-body sensors are connected to the controller unit and are divided into three major classes pertaining to the importance of the location, the sending data significance, and the rate. They are priority-1, priority-2 and priority-3 respectively.

## 5 Scheduling algorithm for charging WSNs

Scheduling based charging algorithms for embedded SNs are designed for both continuous monitoring, and charging implanted sensors before they go below the threshold voltage rating. Performance, along with the data transmission capability of SNs, as per IEEE, are deteriorated below the threshold operated voltage rating. In the suggested algorithm [79], the threshold level is considered as the limiting condition below which the energizing instruction will be activated from the central controller device. Conversely, data loss might happen if it takes additional time to be charged from below the threshold to the nominal voltage level. This delay might cause detrimental effects on human health. Moreover, rechargeable batteries are the power source of SNs in WBANs. Both discharge current and the operating temperature are the influencing parameters in the stored voltage level of SNs. Additionally, voltage behavior is estimated using the energy-aware policies. Non-linear charging and discharging procedures both require time frame to be fully charged. From the node energy profiling and battery kinetic model, the optimal charge storage level is 30% to 70% [80, 81]. Since data transmission is the main issue of WBANs, contention window [42], priority based slack term [56] and biomedical measurement along with energy consumption [82] prefer four (4) cycles to eight (8) cycles bound in the time frame. Especially, the scheduling period for charging the selected SNs is motivated by Table 1 in [56]. For this reason, in-body SNs are activated before six (6) cycles in order to minimize transmission delay. In addition to that, a minimum of three (3) cycles is considered for any SN under the most immediate charging schedule algorithm. On the contrary, both data transmission and sensor charging purpose inductive links may be possible to obstruct one another at the same time. A different frequency level is used to overcome this difficulty for transmission and charging reasons. However, except in an emergency, simultaneous data transmissions and charge procedures are not accepted in this suggested algorithm as nodes will be charged mostly in their inactive state. Combining all the above situations into account, the charging scheme is planned in Algorithm 1.

**Algorithm 1** Scheduling Algorithm to Energize WSNs in WBAN [79]

1: **If** any *V*_*sensor node*_ < *V*_*threshold*_ for next Six (6) cycles

2:  **If** any *V*_*sensor node*_ < *V*_*threshold*_ for next three (3) cycles

3:   Start charging process until nominal voltage level

4:  **Else If** sensor node is not in idle state

5:   wait until *t*_*Data*_ transmitting period

6:   **If** sensor node is not in idle state

7:    wait 2× *t*_*Data*_ transmitting until sensor node idle

8:    **If** sensor node is not in idle state

9:     Go back to Step 1

10:    **End If**

11:   **Else if** *t*_*next Data arrival*_ > *t*_*charging*_

12:    Start charging process until nominal voltage level

13:   **Else**

14:    Start charging until (nominal voltage level ∥ sudden data arrival)

15:    **If** sudden data arrival

16:     wait until *t*_*Data transmitting*_

17:     Go back to Step 11

18:    **End If**

19:   **End If**

20:  **End If**

21: **Else**

22:  Discard the charging scheme

23: **End If**

The charging cycle would be started in the proposed algorithm if the voltage rating is below the threshold condition level. If its existing voltage level can withstand up to the next three (3) cycles, it would be charged on demand based directly. Conversely, the controller will search for the SN inactive situation and calculate the next data arrival period. Considering all these conditions, typical the charging scheme would be started for SN to reach the nominal voltage level. However, to avoid overvoltage problems, when the sensor node voltage reaches the nominal rating, the charging scheme would be stopped.

## 6 Priority-based scheduling algorithm for charging WSNs

The charging scheme mentioned in section 5 is applicable only for a single sensor node. However, in WBAN there are usually many embedded sensor nodes in order to monitor the inside of the human body. Therefore, a proper distribution from the controller unit is the main challenge to allow a regular charging period for the individual sensor nodes. If the proposed scheduling algorithm is applied, there is a major probability of a power crisis for the most important sensor nodes during the data transmission period. To overcome this limitation, a priority aware based CSMA/CA mechanism is applied with the suggested scheduling algorithm to identify the most significant sensor nodes for ensuring a sufficient charge distribution among them. In IEEE 802.15.4, with a fair competition using CSMA/CA before transmission, all sensor nodes try to access the shared channel [43]. However, differentiated service for the sensor nodes is introduced because of the variation in data traffic type and amount. Hence, a CSMA/CA-based priority is suggested to energize different nodes in order to ensure charge distribution among different sensor nodes.

**Algorithm 2** Priority Aware Scheduling Algorithm for Charging WSNs in WBAN

1: Calculate no of nodes, *n* for *V*_*sensor node*_ < *V*_*threshold*_ in next nine (9) cycles

2: **If** *n* > 1

3:  Check decaying rate, *r* for selected *V*_*sensor node*_

4:  Measure sensitivity level, *s* for selected *V*_*sensor node*_ as per CSMA/CA

5:  Label priority, *p* (descending order) as per sensitivity level, *s*

6:  **If** *p*_*sensor,i*_ < *p*_*sensor,j*_; where, *i* ≠ *j*

7:   Follow Algorithm 1 for charging sensor, *i* until next higher priority request

8:   Repeat for *n* sensor nodes

9:  **Else If** *r*_*sensor,i*_ > *r*_*sensor,j*_; where, *i* ≠ *j*

10:   Estimate withstand cycles, *c* for higher decaying rate sensor, *i*

11:   **If** *c* < 2

12:    Follow Algorithm 1 for charging sensor, *i* until next higher priority request

13:    Repeat for *n* sensor nodes

14:   **End If**

15:  **Else If** *p*_*sensor,i*_ < *p*_*sensor,j*_ && *r*_*sensor,j*_ > *r*_*sensor,i*_; where, *i* ≠ *j*

16:   Estimate withstand cycles, *c* for sensor, *i* and sensor, *j*

17:   **If** *c*_*sensor,j*_ < 2

18:    Follow Algorithm 1 for charging sensor, *j* until next higher priority request

19:   **Else**

20:    Follow Algorithm 1 for charging sensor, *i* until next higher priority request

21:   **End If**

22:  **End If**

23: **Else**

24:  Follow Algorithm 1 for charging sensor until next higher priority request

25: **End If**

The priority aware algorithm includes the following three (3) major steps:

**Step 1**: All node voltages have been estimated for the next nine (9) cycles. Here, the time period has been calculated with respect to both Algorithm 1 and the priority aware slack term for the highest priority medical services. Since three (3) cycles have been accepted for the consideration of the emergency charging schedule, the three (3) levels priority leads to the nine (9) cycles charge scheme [56, 82]. Within this cycle, nodes, whose sensor voltage is below the threshold level, has been taken into consideration. Hence, if the number of nodes is more than two (2), proceed to the next step. Otherwise, Algorithm 1 is followed to charge sensor nodes up to the nominal voltage rating.
**Step 2**: This step is applicable for more than one sensor node. A voltage decaying rate of those selected sensor nodes from step 1 has been calculated. Thereafter, the sensitivity level has been measured for those sensor nodes. In this case, the sensitivity of nodes is considered as an input from the user. Priority has been labeled depending on the sensitivity. Usually, implanted sensor nodes have been given more priority due to the placement of a complicated situation inside the human body.
**Step 3**: In this step, charge distribution has been calculated having the information of priority level and voltage decaying rate. Usually, among the selected sensor nodes, a higher priority (lower value of p) has been schemed for charging, as per Algorithm 1. However, a lower priority but fast voltage decaying sensor would be considered if it could not withstand more than two (2) cycles. Here, the decaying rate of two (2) cycles have been calculated as the extreme condition following discharging table [83]. Since our base emergency charging cycle has been calculated three (3) cycles, the decaying rate is considered one (1) cycle less than the emergency. The complicated situation would arise if a lower priority sensor has a higher decaying rate among multiple sensor nodes. In this case, the algorithm is formulated for lower priority sensor nodes that could not cope with more than two (2) cycles to be schemed and energized as per Algorithm 1. This priority scheme would be repeated up to the number of the selected sensor nodes.

In Algorithm 2, a priority followed the CSMA/CA technique is proposed to charge different sensor nodes in WBAN. In the simulation, three sensor nodes have been considered to show the difference for the saturating class—C as well as the class—E driver circuits. It has been proved that the power requirement of a multi-node system has been improved after utilizing the priority-based scheduling for charging the considered nodes.

## 7 Simulation results and performance analysis

To improve the wireless charging performance, a priority-based algorithm has been applied in a scheduled way to a small scale WPT circuit. The proposed wireless charging algorithm for implanted SNs in WBAN has been designed as per carrier sense multiple access with collision avoidance (CSMA/CA) technique. Because of this, the overall power consumption of SNs for certain operation periods, successful charging probabilities for multiple SNs, and instantaneous power requirements have been considered as key performance measures of analysis. It is assumed that proper energy storage in both transmitters and receivers can handle channel interference and traffic contention. The proposed algorithm has been implemented in MATLAB as a simulation environment in order to calculate the circuit components. The suggested algorithm has been justified by circuits composed of saturating class—C with a double along with single MOSFET configured drivers respectively. Traditional, as well as L_*choke*_ modeled class—E configured drivers have been used respectively. A SPICE simulator has also been implemented to collect data generated from different SNs in order to analyze the power consumption for the priority-based scheduling algorithm.

The designed driver circuits have been tested for a system verification in the compact WPT range for WBAN. Although driver circuit selection is distinct in the primary side, the secondary load has been assumed to be the same. Here, secondary output is assumed as telemetry and a non-conductive solenoid which is 6 mm long and 20 mm broad. It has been placed deeply (around 70 mm) inside the patient body. The operation rating of this implanted secondary device is 4 V-regulated supply and 4 mA maximum current.

### 7.1 Numerical results for saturating class—C modeled driver circuit

Saturating class—C driver circuit parameters have been calculated using basic electromagnetic induction equations in [79, 84]. The values of circuit parameters have been presented in Table 1.

**Table 1:** Saturating class—C modeled driver circuit data [79, 84].

Name & Symbol of Parameters	Value	Unit
Primary coil inductance, $L_{s_{1}}$	1.73	uH
Secondary coil inductance, $L_{s_{2}}$	1.73	uH
Primary L-C adjusted coil capacitance, C_1_	14.55	nF
Noise checking capacitance, C_2_	14.65	nF
Designed load resistance, R_*load*_	1125	Ohm
Circuit frequency, f	1	MHz
Link coupling factor, k	0.71	%

**Fig 2:**
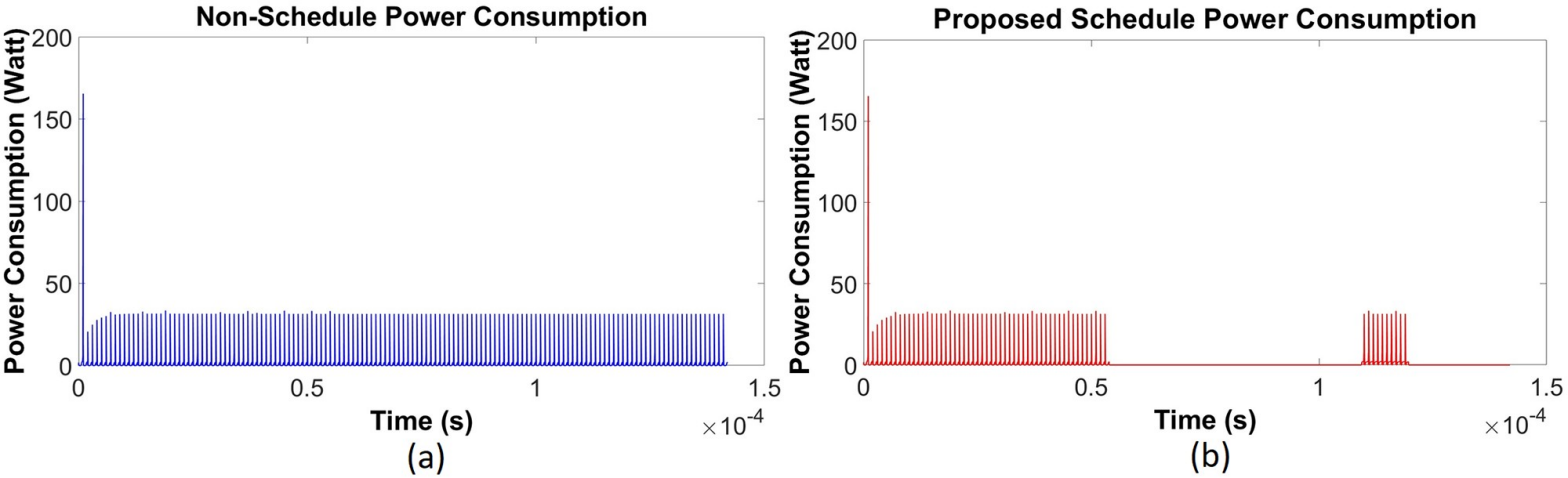
Instantaneous energy consumption for saturating class—C (double MOSFET configured) driver circuit [79]. (a) Existing non-schedule technique (b) Priority aware schedule algorithm.

**Fig 3:**
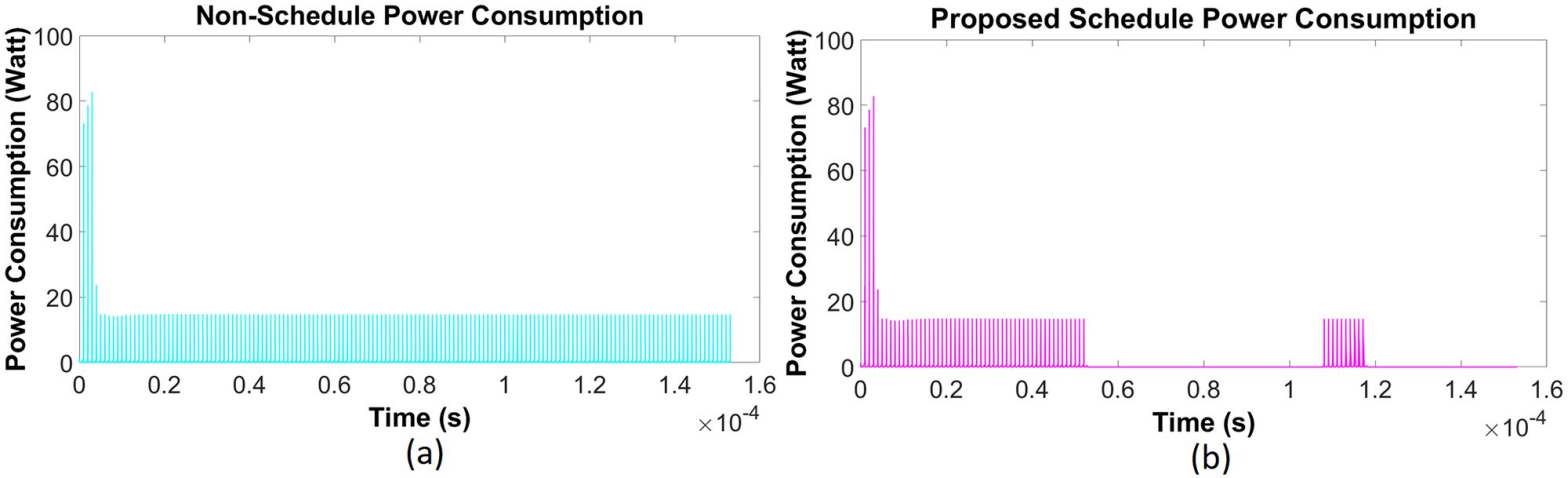
Instantaneous energy consumption for saturating class—C (single MOSFET configured) driver circuit [79]. (a) Existing non-schedule technique (b) Priority aware schedule algorithm.

**Fig 4:**
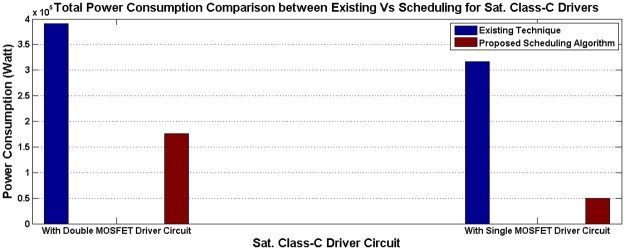
Overall energy consumption comparison between existing and scheduling-based algorithm for saturating class—C modeled driver circuit configurations [79].

**Fig 5:**
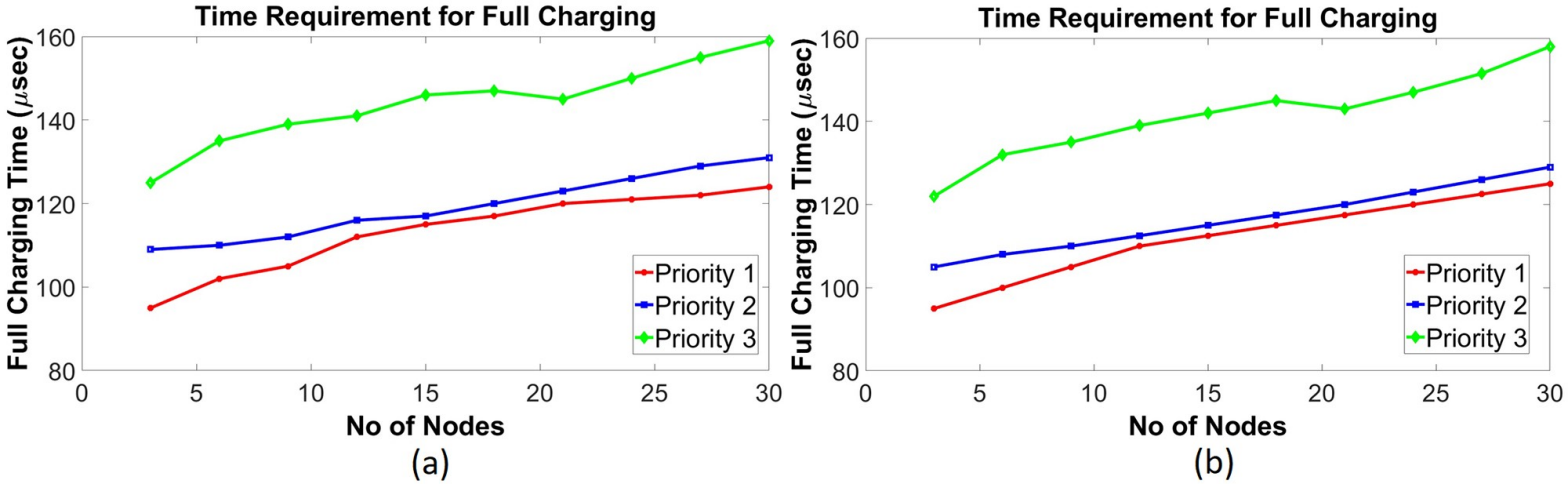
Requirement of full charging time in saturating class—C modeled driver circuits for multiple priority SNs. (a) Double MOSFET (b) Single MOSFET.

Using data given in Table 1, a simulation has been done in the saturating class—C for an existing non-schedule technique and a priority-based proposed scheduling algorithm. Since performance has been measured with respect to the power consumption, multiple time instantaneous power utilization for existing non-schedule (Figs 2a and 3a) and proposed schedule (Figs 2b and 3b) have been shown in Figs 2 and 3 for double and single MOSFET driver circuits respectively. Simulation has been continued for 160 *μ*sec to calculate comparison for overall power consumption between existing and schedule algorithm for different MOSFET configured driver circuits (Fig 4). Moreover, the effect of priority on full charging time in a multi-node system has been predicted in Fig 5a and 5b for double and single MOSFET designed driver circuits respectively.

### 7.2 Numerical results for class—E modeled driver circuit

Basic electromagnetic inductive equations have been followed to calculate the modeling parameters of the class—E driver circuit [85]. The component values have been calculated and shown in Table 2.

**Table 2:** Class—E modeled driver circuit data [85].

Name & Symbol of Parameters	Value	Unit
Primary coil inductance, $L_{s_{1}}$	54.94	uH
Secondary coil inductance, $L_{s_{2}}$	1.73	uH
Choke coil inductance, L_choke_	0.0995	mH
Primary series capacitance, $C_{1_{\text{ser}}}$	426.0	pF
Primary parallel capacitance, $C_{1_{\text{par}}}$	4.72	nF
Primary L-C adjusted coil capacitance, C_2_	14.55	nF
Noise checking capacitance, C_3_	20	nF
Designed load resistance, R_*load*_	1125	Ohm
Circuit frequency, f	1	MHz
Link coupling factor, k	0.7	%

**Fig 6:**
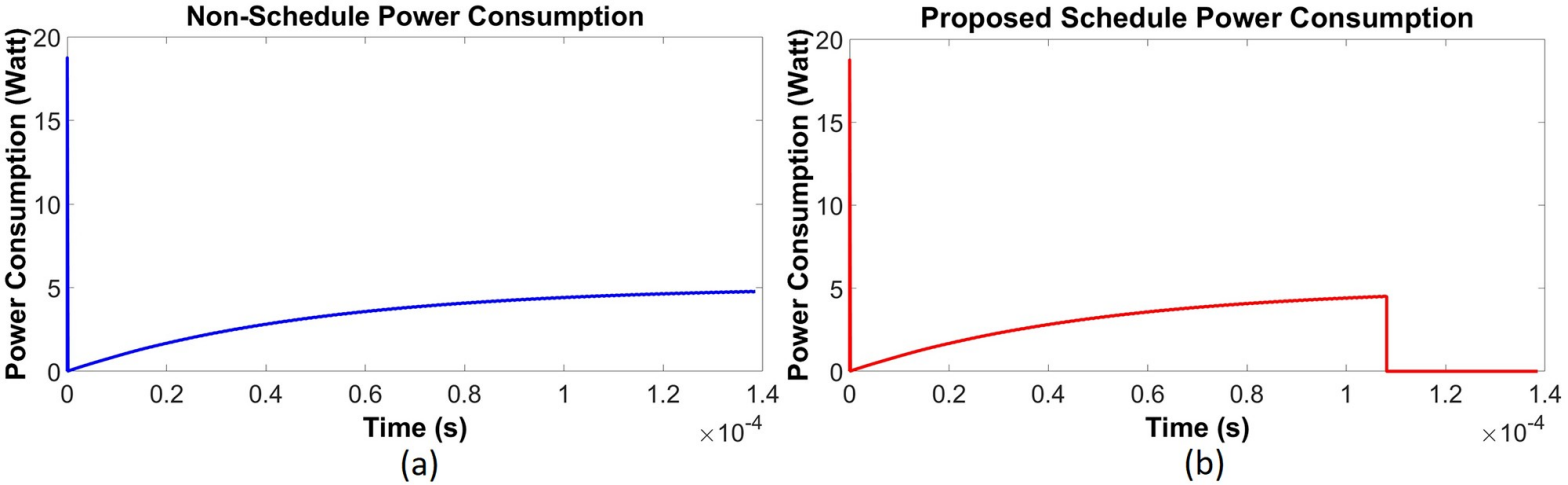
Instantaneous power consumption for class—E (traditional) driver circuit. (a) Existing non-schedule technique (b) Priority aware schedule algorithm.

**Fig 7:**
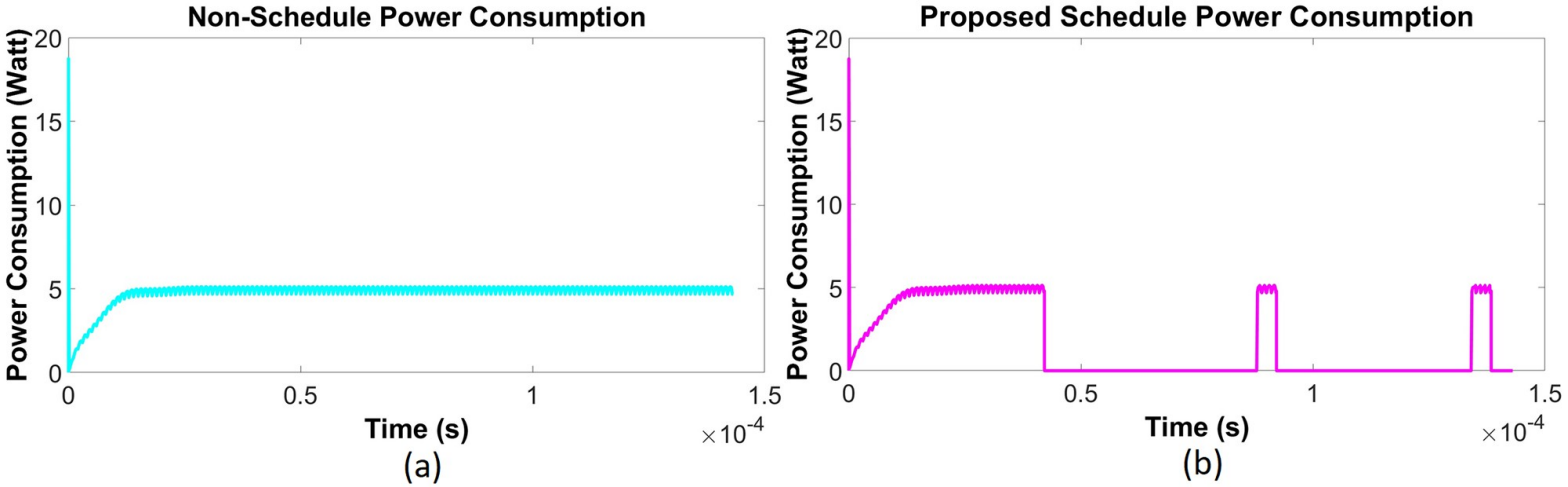
Instantaneous power consumption for L_*choke*_ modeled class—E driver circuit. (a) Existing non-schedule technique (b) Priority aware schedule algorithm.

**Fig 8:**
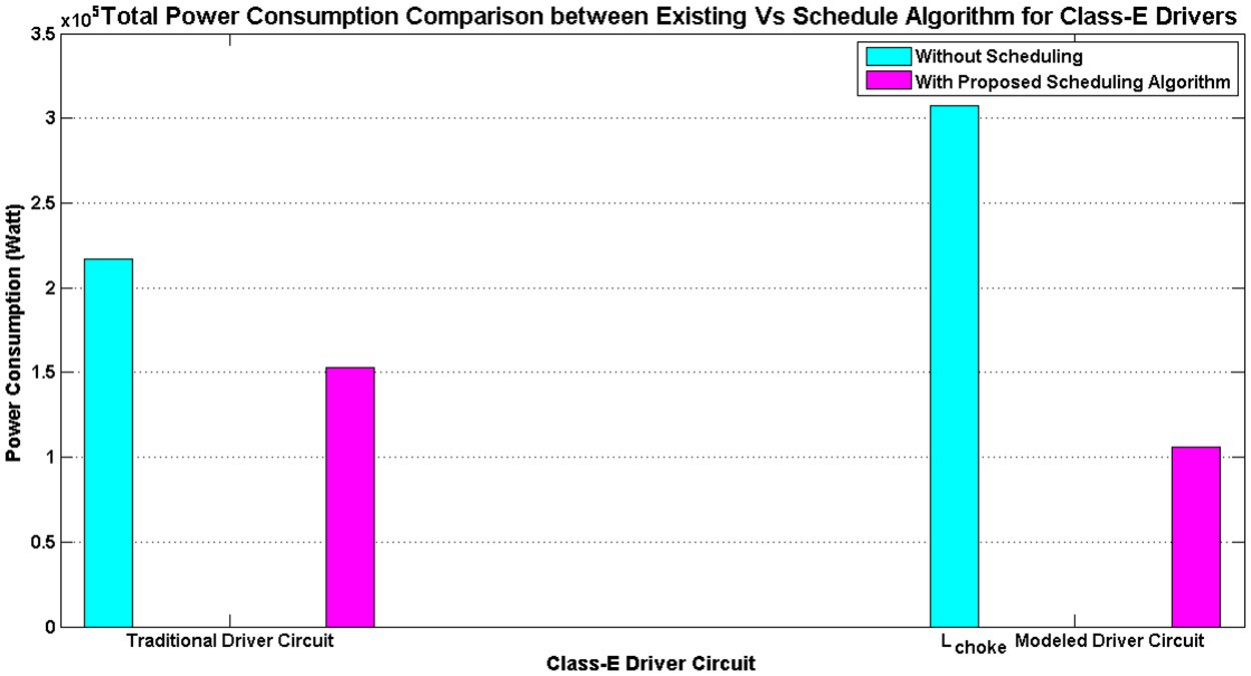
Overall energy consumption comparison between existing and scheduling-based algorithm for class—E modeled driver circuit configurations.

**Fig 9:**
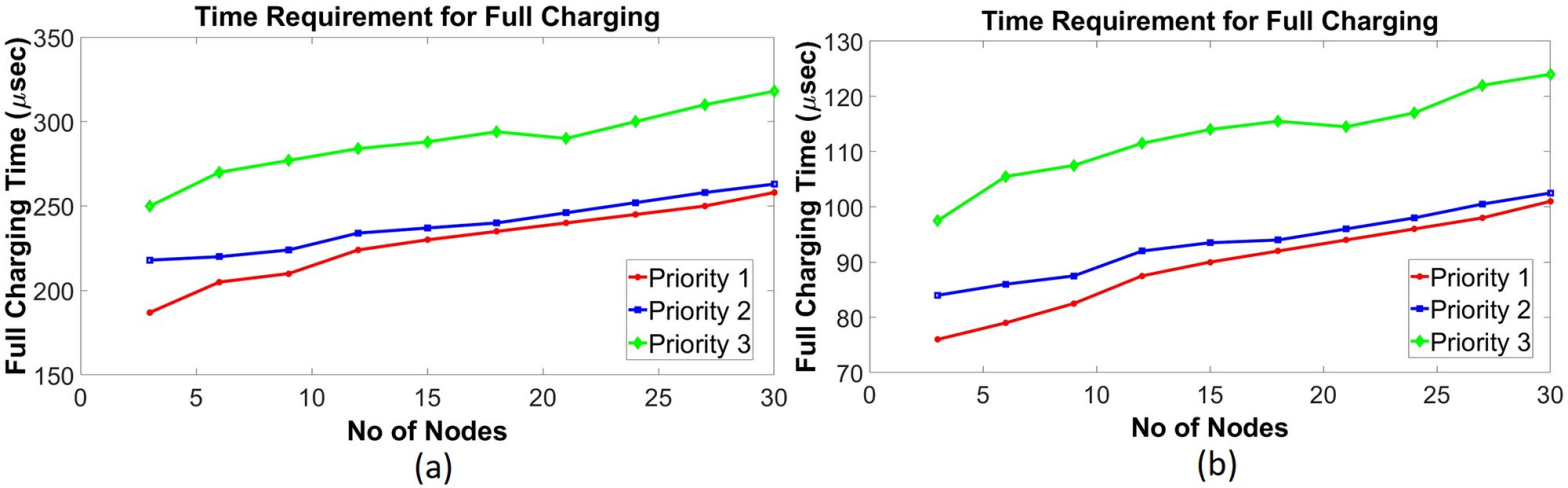
Requirement of full charging time in class—E driver circuits for multiple priority nodes. (a) Without L_*choke*_ modeled (traditional) (b) L_*choke*_ modeled.

Simulation has been done for the existing non-schedule technique and the priority-based proposed schedule algorithm using the data from Table 2 for the class—E driver circuit. Performance of the existing (Figs 6a and 7a) and proposed priority algorithm (Figs 6b and 7b) has been simulated for instantaneous power consumption shown in Figs 6 and 7 for the existing class—E and the L_*choke*_ modeled class—E driver circuits respectively. Total power consumption between these two circuits has been compared in Fig 8 for 140 *μ*sec. The effect of charging time for different priority multi-nodes has been plotted in Fig 9a and in Fig 9b for the existing class—E and the L_*choke*_ modeled class—E associated driver circuits respectively.

### 7.3 Result analysis

The central monitoring unit has been given the required instruction through the collision-free CSMA/CA followed the MAC protocol to the primary of embedded sensor nodes. Subsequently, using induction-based wireless power transfer from the primary to the secondary, necessary operation rating for the embedded sensor nodes have been maintained. For analysis purpose, the primary circuit source has been set as 21.4 volt for the saturating class—C modeled driver circuits and 10 volts for the class—E associated driver circuits. In this research, the priority aware scheduling algorithm has been considered for charging the multi SNs system.

A primary, on body device has been kept running for the whole time, as per the existing technique, in order to meet the requirement of an uninterrupted power supply to the secondary unit. Therefore, a simulation has been done for a specific period of time in order to make a comparison between the prevailing technique and the priority aware scheduling algorithm. After doing the simulation for 142 *μ*sec using the existing technique, the total power consumption for both the double MOSFET and the single MOSFET configured saturating class—C driver circuit has been calculated to be 0.39 MW and 0.32 MW respectively (Fig 4). However, to understand the effect of scheduling, the proposed algorithm has been applied through a central, on body device in order to reduce the overall power consumption for the same operation cycle to 0.176 MW and 0.048 MW respectively (Fig 4). A similar situation has been observed for the traditional class—E and L_*choke*_ modeled class—E driver circuits as well. After utilizing the existing technique, total power consumption during 139 *μ*sec for the above circuits is 0.22 MW and 0.31 MW respectively (Fig 8). Eventually, after going through the proposed algorithm, the total power consumption of the L_*choke*_ modeled class—E driver circuit (0.105 MW) is lower than the traditional one (0.15 MW), although both of them are lower than the existing technique (Fig 8).

The reasons for the reduced power consumption can be explained by Figs 2 to 3 and Figs 6 to 7. Here, to establish the continuous power supply, the existing technique follows uninterrupted operation. Therefore, the sensor node voltage has the probability to be overfed. In the case of the multi-node operation, since priority is not fixed, power distribution is not appropriate for the requirement of demand. Therefore, there is a high possibility to occur underfeeding with some sensor nodes. For these reasons, power consumption is comparatively higher in the existing technique. On the contrary, the proposed algorithm instructs the controller unit through the CSMA/CA mechanism, using the proposed algorithm to operate below the threshold voltage level. Since the primary is not operating continuously, there is less scope to be overfed. Simultaneously, by monitoring through the CSMA/CA mechanism, the scheduling based algorithm has been activated to operate the primary unit circuit by providing the necessarily required power if the SN voltage has been detected to be under 3.6 volts. In addition to the multi-node case, since the controller is not continuously operating, it can take into consideration the other sensors in the off period. Moreover, following the proposed algorithm, the set-up priority will secure the required power distribution to all sensor nodes using the WPT technique. The reason of high spike during start-up for the transient period (Figs 2, 3, 6 and 7) is due to the inrush inductive circuit current. As the duration of those inrushes is less than *msec*, they do not have a significant impact on WBAN and so have been neglected.

Since SN, which is considered as a load, is directly connected to energy consumption, the loss of network energy rises with the increase of SNs. Moreover, the probability of collision causes an increase in energy consumption. In all, energy consumption of different priority SNs increases with the number of nodes having a different preference, due to the network operation. Furthermore, the priority level of the nodes is not the same for multi-nodes system. As a result, the power consumption of SN with a low preference is continually more than the SN with a high priority. Because, in the proposed algorithm, access possibility of SN with high preference is more than SN with low precedence. Since nodes with higher priority have been monitored very frequently, the required charging period is less than the lower priority. In the simulation, three priority levels have been considered. Here, priority 1 has been assumed as the higher priority and priority 3 has been accepted as a lower priority. From Fig 5, it has been observed that priority 1 level nodes require a shorter charging period than priority 3 level nodes for saturating class—C driver circuits (double and single MOSFET configured). This is because, in order to achieve the fixed voltage level, the lesser priority nodes require more time to overcome the contention process and wait for higher priority nodes charging time. A similar situation has been noticed for both the traditional and L_*choke*_ modeled class—E driver circuits as well. From Fig 9, it has been shown that the priority node-1 requires less time for both cases. Whereas, the priority-2 and priority-3 nodes require more time respectively for full charging.

**Fig 10:**
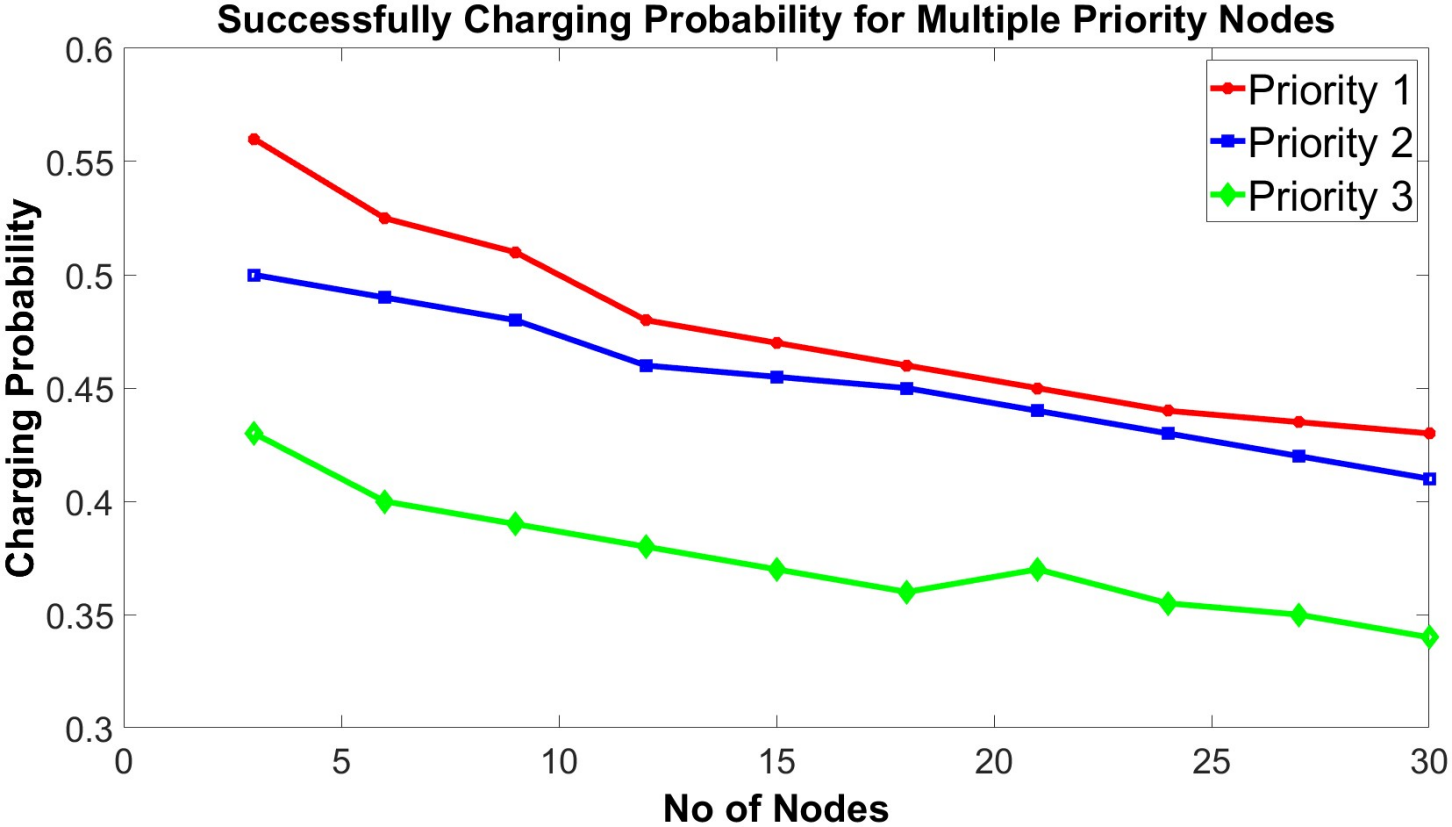
Successful charging probability.

Another interesting observation is the access probability. Since higher priority nodes have been tracked more often than lower priority nodes, the access probability of those nodes is also high. A higher access probability of nodes leads to a higher successful charging possibility. Moreover, as the number of SNs increases, the access probability of all SNs decreases due to the higher contention. From Fig 10, it has been found that priority 1 (higher) level nodes have more successful charging probability than priority 2 (lower than priority 1) and priority 3 (lower priority 2) level nodes. Additionally, keeping the priority level the same, the access probability of all SNs decreases with the increase of node number.

## 8 Conclusion

Energy harvesting by means of wireless power transfer (WPT) has the potential of replacing existing wired based charging schemes with wireless charging, involving the charging of embedded tiny sensor nodes in a wireless body area network (WBAN) environment, which would allow for greater mobility of patients. Such replacement can dramatically improve a patient’s quality of life by allowing them more flexibility of movement and less intensive monitoring due to the non-exposure of embedded sensor nodes to the outside world. In this research work, a novel energy harvesting scheme has been proposed for recharging the power constrained micro sensor nodes that are embedded in sophisticated organs and nervous system inside the human body. The performance of two modified types of WPT drivers previously proposed by us in different literature, namely enhanced saturating class—C driver and class—E driver, to prove their sufficiency for recharging WBAN sensor nodes. Simulation results show that employment of such drivers can sufficiently recharge the nodes in WBAN for up to 70mm distance for 1 MHz system frequency. We have also proposed a priority aware scheduling algorithm for recharging the embedded micro-sensors to ensure the availability of more sensitive and time-critical sensor nodes. Different priority levels have been assigned to different micro-sensors based on the necessity and sensitivity of their functions. Deployment of such algorithms can significantly improve the longevity of more sophisticated nodes. Additional simulation results demonstrate that the required time for full recharging is directly proportional to the number of sensor nodes and higher priority nodes require much less time compared to the nodes with lower priority. Furthermore, successful charging probability is inversely proportional to the number of sensor nodes and higher priority nodes have more successful recharging probability than the lower priority nodes.

## Supporting information

S1 Dataset Saturating class—C (double MOSFET) driver circuit generated dataset. Using the circuit parameter described in Table 1 along with the double MOSFET configuration is employed to generate this dataset. Fig 2 is plotted using this dataset.
(CSV)

S2 Dataset Saturating class—C (single MOSFET) driver circuit generated dataset. The source and load side rating are generated using the single MOSFET driver circuit. Parameters in Table 1 are used to create this dataset. This dataset is applied to generate Fig 3.
(CSV)

S3 Dataset Class—E driver circuit generated dataset. The parameter values of this dataset is calculated using Table 2. Fig 6 is generated using this dataset.
(CSV)

S4 Dataset *L*_*choke*_ modeled class—E driver circuit generated dataset. This dataset is generated using Table 2 of *L*_*choke*_ modeled class—E driver circuit. Fig 7 is plotted using this dataset.
(CSV)
